# Heterogeneity in Macrophage Phagocytosis of *Staphylococcus aureus* Strains: High-Throughput Scanning Cytometry-Based Analysis

**DOI:** 10.1371/journal.pone.0006209

**Published:** 2009-07-10

**Authors:** Glen M. DeLoid, Timothy H. Sulahian, Amy Imrich, Lester Kobzik

**Affiliations:** 1 Molecular and Integrative Physiological Sciences Program, Department of Environmental Health, Harvard School of Public Health, Boston, Massachusetts, United States of America; 2 Department of Pathology, Brigham and Women's Hospital, Boston, Massachusetts, United States of America; Charité-Universitätsmedizin Berlin, Germany

## Abstract

Alveolar macrophages (AMs) can phagocytose unopsonized pathogens such as *S. aureus* via innate immune receptors, such as scavenger receptors (SRs). Cytoskeletal events and signaling pathways involved in phagocytosis of unopsonized bacteria likely govern the fate of ingested pathogens, but are poorly characterized. We have developed a high-throughput scanning cytometry-based assay to quantify phagocytosis of *S. aureus* by adherent human blood-derived AM-like macrophages in a 96-well microplate format. Differential fluorescent labeling of internalized vs. bound bacteria or beads allowed automated image analysis of collapsed confocal stack images acquired by scanning cytometry, and quantification of total particles bound and percent of particles internalized. We compared the effects of the classic SR blocker polyinosinic acid, the cytoskeletal inhibitors cytochalasin D and nocodazole, and the signaling inhibitors staurosporine, Gö 6976, JNK Inhibitor I and KN-93, on phagocytosis of a panel of live unopsonized *S. aureus* strains, (Wood, Seattle 1945 (ATCC 25923), and RN6390), as well as a commercial killed Wood strain, heat-killed Wood strain and latex beads. Our results revealed failure of the SR inhibitor polyinosinic acid to block binding of any live *S. aureus* strains, suggesting that SR-mediated uptake of a commercial killed fluorescent bacterial particle does not accurately model interaction with viable bacteria. We also observed heterogeneity in the effects of cytoskeletal and signaling inhibitors on internalization of different *S. aureus* strains. The data suggest that uptake of unopsonized live *S. aureus* by human macrophages is not mediated by SRs, and that the cellular mechanical and signaling processes that mediate *S. aureus* phagocytosis vary. The findings also demonstrate the potential utility of high-throughput scanning cytometry techniques to study phagocytosis of *S. aureus* and other organisms in greater detail.

## Introduction

*Staphylococcus aureus* is an increasingly frequent and dangerous cause of both community and hospital-acquired pneumonia [1]. The alveolar macrophage (AM) is the primary resident defender against inhaled pathogens such as *S. aureus* that reach the alveoli. In the absence of specific opsonizing antibodies, the AMs ability to clear these pathogens depends upon its innate immune receptors for binding and internalization. In particular, the group of molecular pattern recognition receptors (PRR) collectively known as scavenger receptors (SRs) function in the uptake of unopsonized bacterial pathogens [2]–[9]. SRs are a diverse group of receptors with broad and overlapping specificities that mediate binding and internalization of many polyanionic ligands, including bacteria and their cell wall components, oxidized low density lipoproteins, environmental dusts, apoptotic cells, and CpG DNA. To date, eight classes of SA (A-H) have been described (reviewed in [10]). The most extensively studied in terms of their potential roles in host defense against S. aureus are the class A scavenger receptors SR-AI/II, and macrophage receptor with collagenous structure (MARCO). SR-A I/II binds both Gram-positive and Gram-negative bacteria and their respective cell wall products lipoteichoic acid and lipopolysaccaride [3], [6], [11]. Investigators have used a convenient fluorescence-labeled S. aureus to observe its SR-AI/II-mediated phagocytosis by SR-A transfected CHO cells as well as by human bone-marrow derived macrophages [6]. Moreover, SR-A deficient mice have diminished clearance of *S. aureus* and survival following IP infection, and macrophages from SR-A deficient mice exhibit diminished phagocytosis of *S. aureus* [5]. Another class A SR, MARCO, also binds *E. coli* and *S. aureus*, and this binding is blocked by anti-MARCO antibody, as well as by the general SR binding inhibitors polyinosinic acid and polyguanylic acid [2], [8], [12]. Other SRs that have been implicated as potential mediators of unopsonized pathogen phagocytosis by macrophages include the class A scavenger receptor with C-type lectin (SRCL), also known as collectin placenta-1 (CL-P1) [13]–[15], the class E lectin-like oxidized low-density lipoprotein receptor (LOX-1) [16]–[19], and the class G scavenger receptor that binds phosphatidylserine and oxidized lipoprotein (SR-PSOX), also known as CXC chemokine ligand 16 (CXCL16) [20], [21].

The binding of pathogen ligands or opsonins by macrophage surface receptors initiates signaling cascades. These, in turn, that activate the cytoskeletal machinery responsible for endocytosis and (possibly) for cell activation responses. The signaling events and cytoskeletal elements and activities involved in FcγR-mediated and CR-mediated phagocytosis have been fairly well characterized [22]–[34]. We have previously used a scanning cytometry assay to analyze macrophage phagocytosis of unopsonized latex beads. This study revealed that several cytoskeletal and signaling components previously implicated in FcγR-mediated phagocytosis (tyrosine kinases, protein kinase C (PKC), in complement-mediated phagocytosis (microtubules), or in both (actin, phosphoinositide 3-kinase (PI3K)), as well the MAPK family members JNK and MEK/ERK, are important factors in macrophage uptake of unopsonized albumin-coated latex beads [35].

To allow similar analysis of unopsonized phagocytosis of specific bacterial pathogens we have developed high-throughput fluorescent scanning cytometry-based methods for quantification of phagocytosis of *S. aureus* by adherent human alveolar-like macrophages in a 96-well microplate format. Methods for differential fluorescent labeling of internalized vs. external bacteria were devised to enable automated image analysis of collapsed confocal stack images from scanning cytometry. Analysis and quantification software was developed to provide enumeration of internalized, bound external and total bacteria per cell, and calculation of mean bacteria per cell and fraction of bacteria internalized within each microplate well. The format made it possible to test a panel of inhibitors of pathways related to phagocytosis. The data confirmed the role of scavenger receptors in binding of the popular commercially available fluorescent S. aureus-derived particles. In contrast, macrophages showed SR-independent uptake of a panel of other S. aureus strains (live and heat-killed). Strain-specific heterogeneity in the effects of signaling inhibitors was also seen.

## Materials and Methods

### Human cell isolation and preparation

Human peripheral blood monocyte-derived, granulocyte and macrophage-colony stimulating factor (GM-CSF)-matured MФs (GM-MФs) were prepared as previously described [35]. Briefly, buffy coats harvested from blood obtained from discarded platelet apheresis collars obtained from the Kraft Family Blood Donor Center at the Dana Farber Cancer Institute (Boston, MA, USA) were enriched for monocytes using the RosetteSep Monocyte Enrichment kit (Stem Cell Technologies, Vancouver, BC, Canada). Monocytes were cultured in Vuelife bags (American Fluoroseal, Gaithersburg, MD, USA) at 5% CO_2_ and 37°C in RPMI/10% FBS with 20 µg/ml gentamycin and 20 ng/ml human recombinant GM-CSF (Peprotech, Rocky Hill, NJ, USA). For adherent cell studies harvested cells were resuspended at 5×10^5^/ml in RPMI/10% FBS and dispensed into black-walled 96 well Micro-Clear plates (Greiner Bio-One, Monroe, NC, USA) at 1×10^5^ cells/well, and incubated for 48 hours at 5% CO_2_ and 37°C. For suspension cell flow cytometry studies harvested cells were resuspended at 5×10^5^/ml in RPMI/ 0.3% BSA and stored on ice until time of assay (less than one hour).

### Mouse cell isolation and preparation

Animal experiments and husbandry were approved by the Harvard Medical Area Standing Committee on Animals. 4–6 week-old female BALB/c mice (Jackson Laboratory, Bar Harbor, ME, USA) were injected i.p. with 2 ml aged thioglycolate broth (Sigma-Aldrich, St. Louis, MO, USA). Four days post injection mice were anesthetized with isofluorane and euthanized by cervical dislocation. Peritoneal lavage was performed with 10 ml. ice-cold PBS and pooled samples from three mice were centrifuged at 500× g for 8 minutes. Cells were resuspended at 5×10^5^/ml in RPMI/10% FBS and dispensed into black-walled 96 well Micro-Clear plates (Greiner Bio-One, Monroe, NC, USA) at 1×10^5^ cells/well, and incubated for 48 hours at 5% CO_2_ and 37°C.

### Preparation of live *S. aureus* suspensions

*S. aureus* strains Seattle 1945 (ATCC 25923), Wood, and ATCC 12598 (Cowan 1) were purchased from American Type Culture Collections (ATCC, Manassas, VA, USA). Green fluorescent protein (GFP)-expressing *S. aureus* strain RN6390 was generously donated by Dr. Ambrose Cheung, Dartmouth Medical School, Hanover, NH, USA). 300 µL of thawed −80°C frozen *S. aureus* stocks were added to 10 ml of Tryptic Soy broth (with 2.5 µg/ml chloramphenicol selection agent for GFP+ RN6390) in 50 ml conical centrifuge tubes, and incubated at 37°C while shaking at 250 rpm for four hours. Bacteria were washed twice in cold PBS and resuspended in cold PBS, vortexed for two minutes and passed through a 5 µm syringe filter. OD 650 was used to estimate CFU from standard curves. For unopsonized experiments bacteria were suspended in cold RPMI/.3%BSA without serum or phenol red (assay buffer) at 2.67×10^8^/ml and kept on ice until infection of cells. For IgG-opsonized experiments bacteria were diluted to 4×10^8^/ml in assay buffer, incubated on ice for 30 minutes with 5 µg/ml mouse monoclonal IgG_3_ anti-*S. aureus* antibody (C55570M, Biodesign International, Saco, ME, USA), washed twice in cold PBS, resuspended in assay buffer at 4×10^7^/ml, and kept on ice until infection of cells.

### Preparation of heat-killed *S. aureus* Wood strain suspension

Live *S. aureus* Wood strain were prepared as described above. Following OD 650 determination bacteria (suspended in PBS) were incubated in a water bath heated to 63°C for 30 minutes, cooled on ice, centrifuged and resuspended in assay buffer at 2.67×10^8^/ml and kept on ice until infection of cells.

### Preparation of commercial killed *S. aureus* Wood strain suspension

Lyopholized AlexaFluor 488-conjugated heat or irradiation killed Wood strain *S. aureus* (Life Technologies, Carlsbad, CA, USA) was suspended in cold assay buffer at a concentration of 1.33×10^7^/ml and sonicated for 20 seconds immediately prior to use.

### Phagocytosis assay: latex beads

All reagents and buffers were at room temperature when added to cells, and all incubations were at 5% CO_2_ and 37°C. Adherent GM-MФs prepared as described above were incubated with 100 µM CellTracker Blue CMAC (Life Technologies) in HBSS with Ca^++^ and Mg^++^ for 40 minutes at 5% CO_2_ and 37°C, followed by replacement with RPMI/.3%BSA without serum or phenol red (assay buffer). Inhibitors or control solutions (table 1) were added to cells (with triplicate wells for each condition) 40 minutes prior to addition of beads. Polyinosinic acid, chondroitin sulfate, cytochalasin D, nocodazole, and staurosporine were purchased from Sigma-Aldrich (St. Louis, MO, USA). Gö 6976, JNK Inhibitor I, JNK Inhibitor I control, and KN-93 were purchased from EMD Chemicals (San Diego, CA, USA).

**Table 1:** Inhibitors used.

Inhibitor	Target	Control	Concentration	Vehicle (final %)
poly(I)	SR binding	chondroitin S.	10, 100 µg/ml	PBS (1.0, 10.0)
cytochalasin D	actin polymerization	vehicle	15 µM	DMSO (0.15)
nocodazole	microtubule polymerization	vehicle	25 µM	DMSO (0.5)
Staurosproine	ptotein kinases	vehicle	1 µM	DMSO (0.1)
Gö 6976	protein kinase C	vehicle	10 µM	DMSO (0.2)
JNK Inhibitor I	c-Jun N-terminal kinases	inactive analog	4 µM	PBS (0.4)
KN-93	CaM Kinase II	vehicle	20 µM	DMSO (1.0)

For latex bead uptake assays, one micron green fluorescent carboxylated latex beads (Life Technologies) were biotinylated as previously described [35]. Beads were suspended in assay buffer at 2.67×10^8^/ml and sonicated for 20 seconds immediately prior to use. Cells were incubated for 20 minutes with bead suspension at a final concentration of 2×10^8^/ml with inhibitors or controls for binding and internalization, washed three times with HBSS/0.3% BSA, and incubated an additional 20 minutes in assay buffer with inhibitors or controls for further internalization. Microplates were transferred to ice to halt internalization and cells were incubated on ice for 30 minutes with 20 µg/ml streptavidin-Texas Red (Life Technologies) in assay buffer to label external beads. Cells were then washed with HBSS/0.3% BSA and fixed with 4% paraformaldehyde in PBS for 10 minutes. Cells were stored in 1% paraformaldehyde at 4°C overnight. Cells were incubated for 60 minutes at room temperature with 2 µg/ml Hoechst 33342 (Life Technologies) in PBS for nuclear staining and then stored in PBS at 4°C until imaged.

For assays of uptake of green fluorescent *S. aureus*, cells were incubated for 20 minutes with unopsonized GFP+ RN6390 (final concentrations of 2×10^8^/ml), 3×10^7^/ml for IgG-opsonized GFP+ RN6390, or 1×10^7^/ml for AlexaFluor 488-conjugated killed Wood strain) with inhibitors or controls (table 1) for binding and internalization, washed three times with HBSS/0.3% BSA, and incubated an additional 20 minutes in assay buffer with inhibitors or controls for further internalization. Cells were fixed with 4% paraformaldehyde in PBS for 10 minutes. To achieve red fluorescent labeling of external bacteria cells were incubated for 15 minutes with PBS/4% FBS (blocking buffer), incubated for 40 minutes with 10 µg/ml mouse monoclonal IgG_3_ anti-*S. aureus* antibody in blocking buffer, washed three times with blocking buffer, incubated for 20 minutes with 40 µg/ml Texas-RedX-conjugated goat anti-mouse IgG (Life Technologies) in blocking buffer, and washed twice with blocking buffer. Hoechst nuclear staining was then performed as described above.

In some assay validation experiments the phagocytosis assay was performed using the GFP-expressing RN6390 strain and the single inhibitor cytochalasin D (to prevent internalization) or vehicle control, with the exception that prior to fixation and immunolabeling cells were incubated for an additional 60 minutes at 5% CO_2_ and 37°C in assay media with cytochalasin D or control and with or without 10 µg/ml lysostaphin (Sigma-Aldrich) to dissolve external *S. aureus*.

For assays of uptake of non-fluorescent S. aureus strains (Seattle 1945, live Wood or Cowan 1 strain) prelabeling with CellTracker Blue, incubation of macrophages with bacteria, fixation and incubation with primary anti-*S. aureus* antibody was carried out as described above. For dual red and green fluorescent labeling of external bacteria, following primary antibody incubation and three washes with blocking buffer, cells were incubated for 20 minutes with both 40 µg/ml Texas-RedX conjugated goat anti-mouse IgG (Life Technologies) and 40 µg/ml AlexaFluor 488 conjugated goat anti-mouse IgG Fab fragment (Life Technologies) in blocking buffer and then washed twice with blocking buffer. For green fluorescent labeling of internal bacteria cells were permeabilized by incubation for 20 minutes with 0.1% saponin in blocking buffer (BP buffer), then incubated for 60 minutes with10 µg/ml mouse monoclonal IgG_3_ anti-*S. aureus* antibody in BP buffer, washed three times for five minutes each with BP buffer, incubated for 30 minutes with 40 µg/ml AlexaFluor 488 conjugated goat anti-mouse IgG Fab fragment in BP buffer, and washed three times for five minutes each BP buffer. Hoechst nuclear staining was then performed as described above.

### Flow cytometry analysis of particle uptake in suspension

GM-MФs in suspension were prepared as described above. Polyinosinic acid or chondroitin sulfate were added to a final concentration of 10 µg/ml and cells were incubated on ice for 30 minutes. Green fluorescent latex beads, commercial killed AlexaFluor 488-conjugated S. aureus Wood strain and GFP+ S. aureus RN6390 strain suspensions, were prepared as described above (but at 2× concentrations). Volumes of 2× particle suspensions equaling cell suspension volumes were added and cells were incubated for 30 minutes at room temperature. An equal volume of 4% paraformaldehyde was added (final concentration of 2%) and cells were incubated an additional 10 minutes at room temperature for fixation. Cell suspensions were analyzed using a FacsCanto II flow cytometer (BD Biosciences).

### Image acquisition and data analysis

Images were acquired using the BD Pathway 855 High-Content Bioimager and AttoVision imaging software (BD Biosciences, San Jose, CA, USA) with a 20X NA075 lens (Olympus, Center Valley, PA, USA). Green channel images of green fluorescent beads, or of GFP-expressing or AlexaFluor488-labeled *S. aureus* were acquired using 470/40 band-pass (BP) excitation, 515 long-pass (LP) dichroic and 515 LP emission filters, red channel images of Texas Red and Texas-RedX labeled beads or *S. aureus* were imaged using 560/20 BP excitation, 595 LP dichroic and 645 LP emission filters, and blue channel images of Hoechst nuclear and CellTracker Blue cytoplasmic labels were acquired using 380/20 BP excitation, 400 LP dichroic, 435 LP emission filters. Auto-focusing was performed based on Hoechst/CellTracker blue signals. For each of the three channels confocal images (with flat field correction and 2×2 binning) were collected at 1.5 µm intervals along the Z-axis throughout the thickness of the cells, and a single combined collapsed confocal stack image was generated. Three non-overlapping 20X image fields were acquired for each microplate well to produce a total of nine sets of images (3 fields ×3 wells) per condition for each cell donor.

### Image analysis and quantification

Using the collapsed confocal stack images, software was developed to define the cells (blue emission channel image), count the number of beads per cell (green emission channel image) and determine if the beads are outside the cell (red emission channel image) using custom software developed in MATLAB® (The Mathworks, Inc., Natick, MA, USA). Hoechst/CellTracker Blue images were processed to reduce noise, enhance contrast and correct for non-uniform field brightness. A gradient-facilitated watershed segmentation algorithm was used to identify and label individual cells.

Green and red fluorescent bead and bacteria images were sharpened and processed to remove background noise. Watershed segmentation was used to identify and label individual bead/bacteria objects. Labeled bead or bacteria objects within the all particles (green) image channel overlapping (sharing one or more pixel with) a bead or bacteria object in the external particle (red) image channel were classified as “external”, and were otherwise classified as “internal”. Particle objects sharing one or more pixel with any cell object were considered to be associated with that cell.

Typically a total of between 500 and 1000 cells were identified and quantified (from the nine image fields acquired) per donor per condition. Binding for each donor and condition was calculated as the mean number of cell-associated beads or bacteria per cell (total particles/number of cells). The mean fraction of particles internalized by all cells for each donor and condition was calculated from the total numbers of internal and external beads or bacteria (internal/[internal + external]).

For poly(I)-treated cells mean bound particles/cell and mean fraction internalization were normalized to chondroitin sulfate control values, and significant differences were calculated using a Students paired *t*-test. For all other inhibitors treatment and vehicle or inactive analog control values were normalized to those of a null control, and significant differences were calculated using a one-way paired ANOVA with Tukey's multiple comparison of all means. Prism 4 for Windows (Graphpad Software, San Diego, CA, USA) was used for all graphing and statistical calculations.

## Results

### Validation of scanning cytometry-based phagocytosis assay

**Figure 1:**
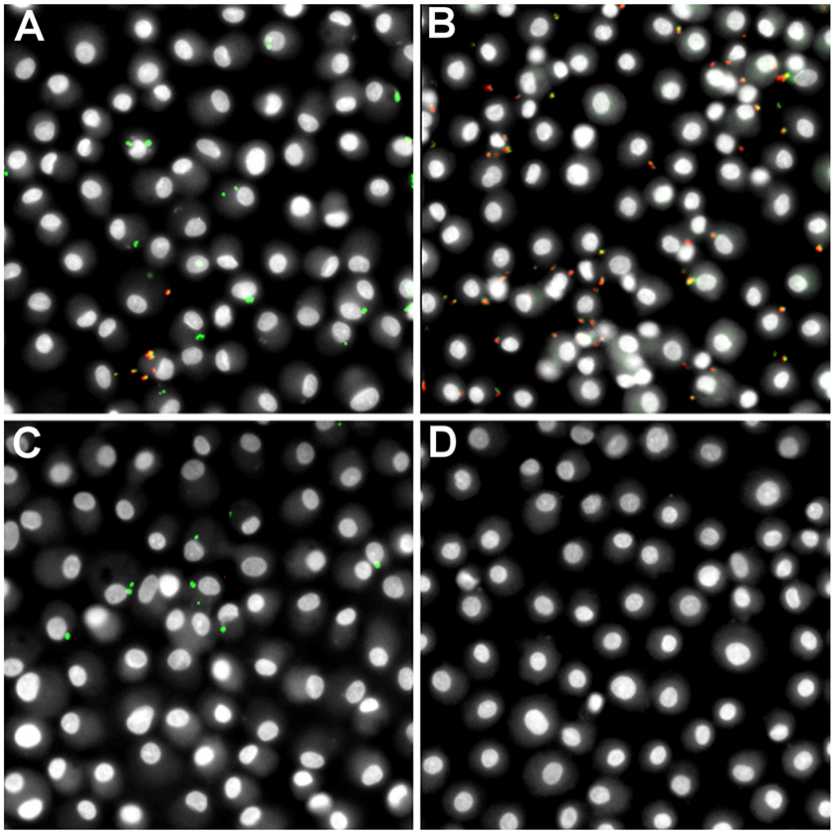
Validation of external *S. aureus* labeling. Adherent GM-MФs were either treated with cytochalasin D (to block uptake) or control prior to and during incubation with GFP-*S. aureus* RN6390 and incubated with lysostaphin (to degrade external *S. aureus*, eliminating external bacteria) or control following incubation with *S. aureus*. External *S. aureus* were labeled with an IgG_3_ monoclonal mouse anti-*S. aureus* primary antibody and Texas-RedX-conjugated goat anti-mouse secondary antibody. (A) No treatment. (B) Treatment with cytochalasin D. (C) Treatment with lysostaphin. (D) Treatment with both cytochalasin D and lysostaphin. (Original magnification, 200X).

We adapted a high-throughput phagocytosis assay, previously developed in our lab for quantification of latex bead phagocytosis [35], for quantification of *S. aureus* phagocytosis. Differential immunolabeling methods were developed for either fluorescent (GFP-expressing) or non-fluorescent *S. aureus* to produce an end result in which all bacteria were green-fluorescent and external bacteria were also red-fluorescent (see methods for details). To test that external labeling of bacteria was effective and limited to external bacteria, GM*-*MФs were incubated with GFP-*S. aureus* RN6390 with or without cytochalasin D treatment (to block internalization), and incubated after bacterial challenge with or without lysostaphin – an enzyme produced by *Staphylococcus staphylolyticus* that lyses other *Staphylococcus* species [36] by hydrolyzing glycylglycine bonds in the polyglycine cross-bridges between glycopeptide chains in the cell wall peptidoglycan of Staphylococcus species [37]. Representative composite images of the four possible combinations are depicted in figure 1. Control cells (no treatment) show a mixture of internalized (green) and external (green+red = yellow/orange) *S. aureus* (figure 1A). Cytochalasin D treatment prevents internalization of bacteria making them accessible to immunolabeling; most bacteria show antibody labeling seen as yellow/orange in composite images (figure 1B). Treatment with lysostaphin alone results in cells with internalized bacteria (green) only (figure 1C). Treatment with both cytochalasin D (blocking internalization) and lysostaphin (degrading external bacteria) results in complete elimination of bacteria (figure 1D).

**Figure 2:**
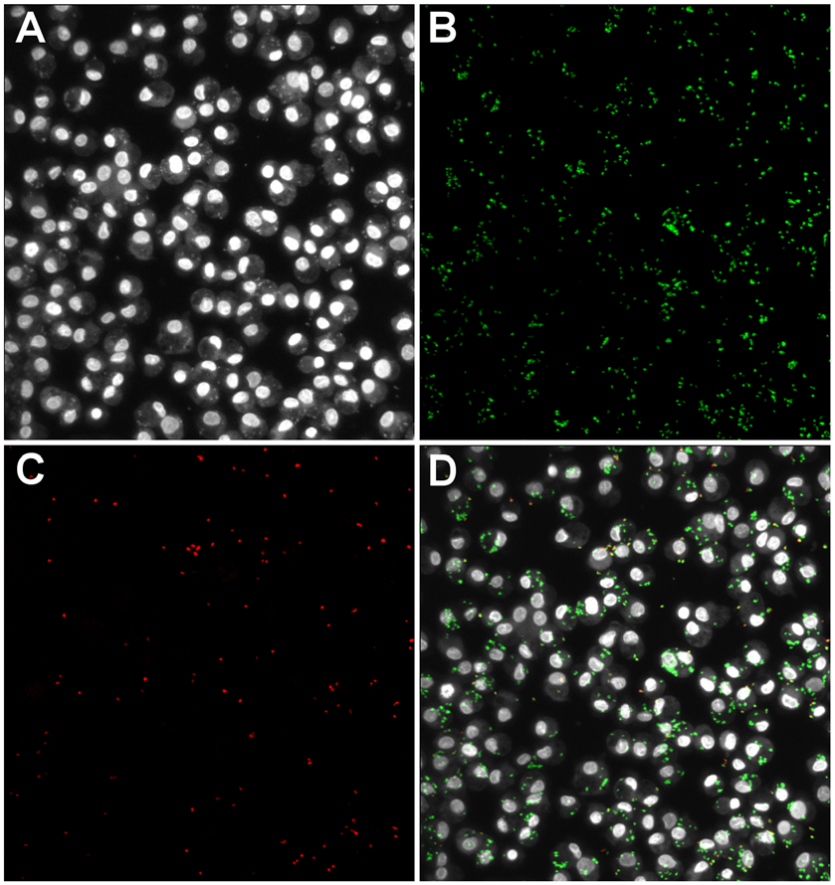
Scanning cytometry fluorescence imaging with GFP-S. aureus RN6390. Adherent GM-MФs were incubated with unopsonized GFP-*S. aureus* RN6390. External *S. aureus* were labeled with an IgG_3_ monoclonal mouse anti-*S. aureus* primary antibody and Texas-RedX-conjugated goat anti-mouse secondary antibody. Collapsed confocal stack images were acquired by scanning cytometry. (A) CellTracker Blue and Hoechst channel (cells). (B) GFP channel (all bacteria). (C) Texas Red channel (external bacteria). (D) Composite image. (Original magnification, 200X).

**Figure 3:**
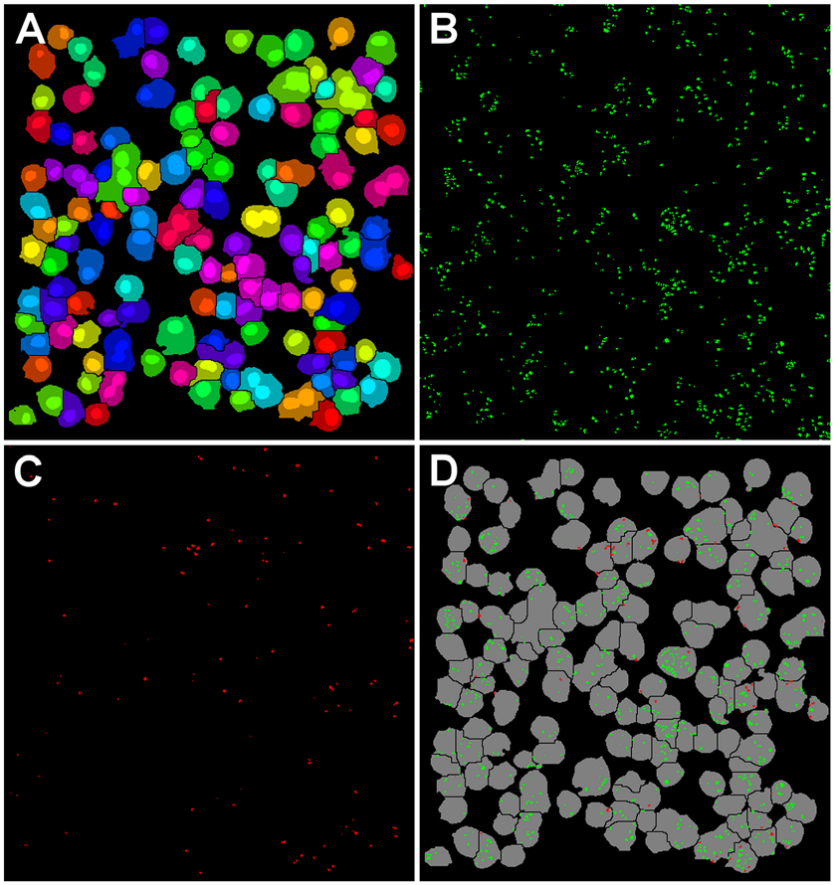
Image processing of scanning cytometry images. Image processing and analysis was performed using custom software developed in MATLAB®. (A) Individual cells segmented and identified in cell image. (B) Individual bacteria segmented and identified in all bacteria image. (C) Individual bacteria segmented and identified in external bacteria image. (D) Internalized (green) and bound but not internalized (red) bacteria assigned to individual cells (gray).

Scanning cytometry was used to acquire confocal image stacks throughout the depth of the adherent cells and generate single collapsed confocal stack images for each of the three color channels (cells, all beads, internal beads) as shown in figure 2. Custom image analysis software was developed in MATLAB® and used to identify individual cell objects, count internalized vs. bound but external beads for each cell (figure 3), and calculate mean total bacteria and percent internalization for all cells in each well. To validate the automated quantification of bound and internalized bacteria we compared the results of automated image analysis to human counting of the same images. Bound and internalized bacteria per cell were manually counted for 50 cells in control and cytochalasin D treated conditions and compared to software quantification of the same 50 cells. Automated counts varied from manual counts by less than 10% for each measure (table 2).

**Table 2:** Comparison of automated vs. manual quantification of phagocytosis.

source	internal bacteria	% diff	external bacteria	% diff	total bacteria	% diff	% internal	% diff
**manual control**	**387**	**-**	**80**	**-**	**467**	**-**	**82.9**	**-**
**automated control**	**371**	**4.1**	**79**	**1.3**	**450**	**3.6**	**82.4**	**0.5**
**manual +cyto D**	**10**	**-**	**132**	**-**	**142**	**-**	**7.0**	**-**
**automated +cyto D**	**11**	**10**	**122**	**7.6**	**133**	**6.3**	**8.3**	**1.3**

Numbers in internal, external and total bacteria columns represent total count for fifty cells.

% internal = 100× (internal bacteria)/(internal bacteria + external bacteria).

% diff: 100× |automated – manual|/manual.

### The SR inhibitor polyinosinic acid does not block binding of most unopsonized *S. aureus*

**Figure 4:**
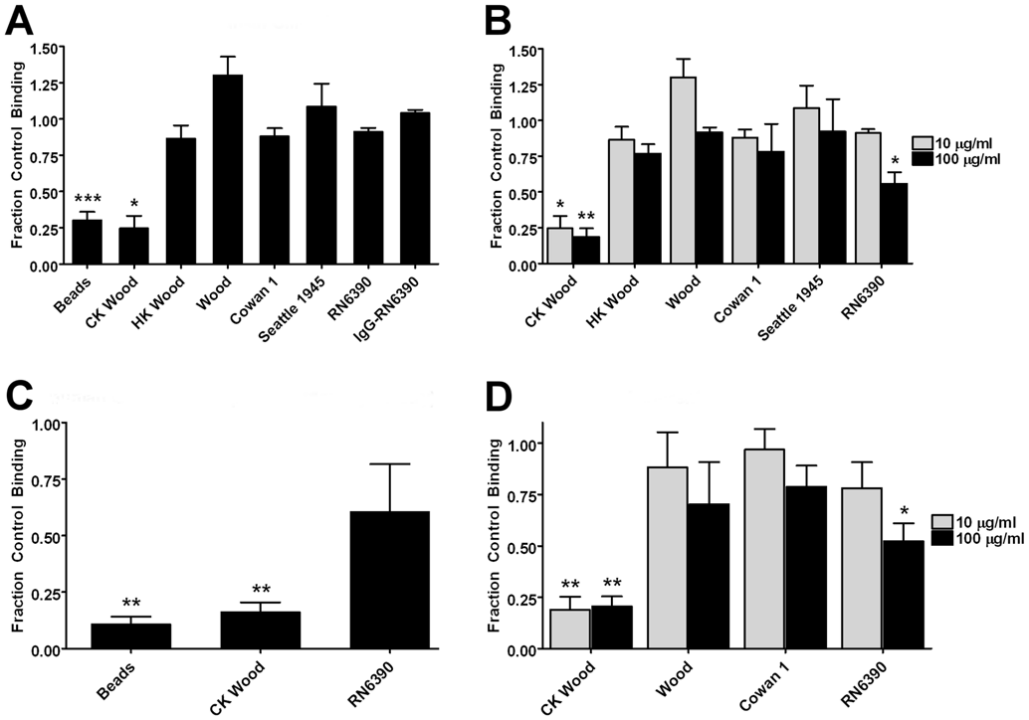
Effect of poly(I) on binding of *S. aureus* strains by macrophages: quantitative results. Macrophages were treated with 10 or 100 µg/ml poly(I) or its negative control chondroitin sulfate prior to and during incubation with either fluorescent latex beads or *S. aureus*. Bars represent the mean of the fraction of chondroitin sulfate control binding [particles per cell for adherent cells (A,B,D) and mean fluorescence increase over that of cells without particles for cells in suspension (C)] of three normal donors±SEM. (A) Results of inhibition by 10 µg/ml poly(I) of binding by adherent GM-MФs. (B) Results of inhibition by 10 vs. 100 µg/ml poly(I) of binding by adherent GM-MФs. (C) Results of inhibition by 10 µg/ml poly(I) of binding by GM-MФs in suspension. (D) Results of inhibition by 10 vs. 100 µg/ml poly(I) of binding by adherent mouse peritoneal macrophages. *p<0.05, **p<0.01, ***p<0.001 compared to negative control. CK Wood = commercial killed AlexaFluor 488-conjugated S. aureus, Wood Strain. HK Wood = heat-killed S. aureus, Wood strain.

**Figure 5:**
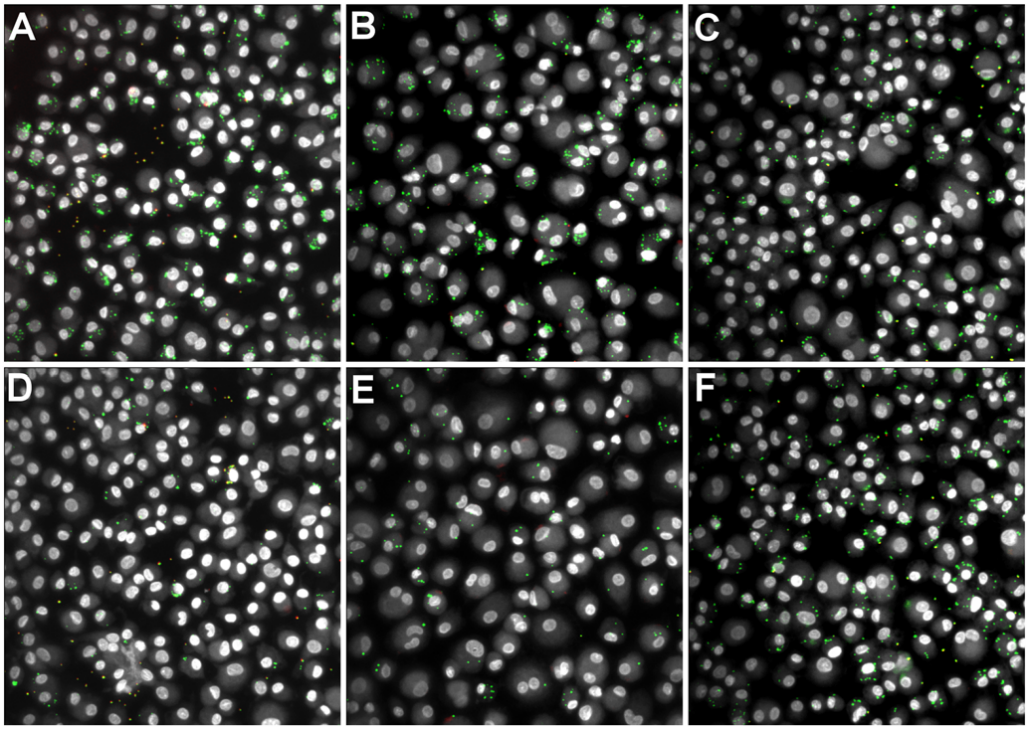
Effect of poly(I) on binding of *S. aureus* strains by adherent GM-MФ: image results. Adherent GM-MФ were treated with 10 µg/ml poly(I) or its negative control chondroitin sulfate prior to and during incubation with either fluorescent latex beads or *S. aureus* (commercial killed AlexaFluor 488-conjugated Wood or live Wood strain). Images are merged composites of cell (gray), internalized beads or bacteria (green) and external beads or bacteria (red). (A,B,C) Chondroitin sulfate control. (D,E,F) Poly(I)-treated. (A,D) Cells with fluorescent latex beads. (B,E) Cells with commercial killed AlexaFluor 488-conjugated Wood strain *S. aureus*. (C,F) Cells with live Wood strain *S. aureus*.

To examine the role of SRs in phagocytosis of *S. aureus*, adherent GM-MФs were treated with the SR blocker poly(I) or the control polyanion chondroitin sulfate prior to and during incubation with a panel of *S. aureus* strains. The assay also tested uptake of unopsonized latex beads, used as a positive control for SR-mediated uptake. Binding and internalization was analyzed by scanning cytometry as described in methods. At 10 µg/ml poly(I) reduced binding of latex beads by 70% compared with control (p<.001) and inhibited binding of commercial killed *S. aureus* Wood strain by 75% of control (p<.05). In contrast, there was no significant effect on binding of unopsonized live or heat-killed Wood, Cowan 1, Seattle 1945 (ATCC 25923) or RN6390 strains, or on that of IgG-opsonized RN6390 (figure 4A). At 100 µg/ml poly(I), minimal increases in the extent of inhibition of binding of latex beads or commercial killed Wood strain were observed, suggesting that maximal effects of this SR inhibitor were already seen at the lower concentration. At this higher concentration, a partial inhibition of binding of unopsonized RN6390 was seen (44%, p<.05). There was no significant effect at this concentration on binding of live or heat-killed Wood, Cowan 1, and Seattle 1945 (figure 4B). Representative composite images of control and poly(I) treated cells after phagocytosis of unopsonized latex beads, commercial killed Wood strain, and live Wood strain are shown in figure 5, and are consistent with the automated analysis results.

**Figure 6:**
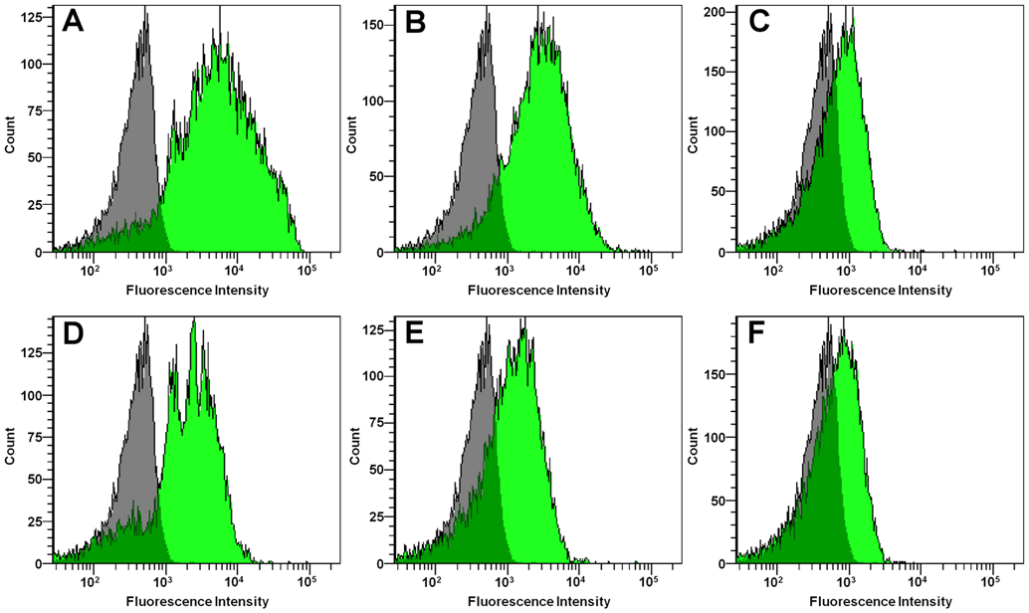
Effect of poly(I) on binding of *S. aureus* strains by GM-MФ in suspension – flow cytometry. Adherent GM-MФ in suspension were treated with 10 µg/ml poly(I) or its negative control chondroitin sulfate prior to and during incubation with either fluorescent latex beads or *S. aureus* (commercial killed Wood or RN6390 strain). Histograms represent green fluorescence intensity counts of 10,000 cells for a single donor. Gray histograms represent cells without particles. Green histograms represent cells with particles. (A,B,C) Chondroitin sulfate control. (D,E,F) Poly(I) treated. (A,D) Cells with fluorescent latex beads. (B,E) Cells with commercial killed AlexaFluor 488-conjugated Wood strain *S. aureus*. (C,F) Cells with GFP+RN6390 strain *S. aureus*.

Since some prior studies used suspension-based assays to observe SR-mediated uptake of fluorescent S. aureus, we compared results using adherent vs. suspended macrophages. GM-MФs in suspension were treated with 10 µg/ml poly(I) or chondroitin sulfate, and incubated with *S. aureus* (commercial killed Wood or RN6390 strain) or green fluorescent latex beads and analyzed by flow cytometry. Poly(I) reduced binding of latex beads by 90% compared with control (p<.01) and inhibited binding of commercial killed *S. aureus* Wood strain by 85% (p<.01), consistent with prior findings [2], [4], [6], [8], [9], [35]. In contrast, there was no significant effect on binding of *S. aureus* RN6390 (figure 4C and figure 6).

We applied a similar analysis to macrophages from a different species and anatomic site, murine peritoneal exudate macrophages. Adherent mouse thioglycolate-elicited peritoneal macrophages were pre-treated with 10 or 100 µg/ml poly(I) or chondroitin sulfate and incubated with *S. aureus* (commercial killed or live Wood, Cowan 1 or RN6390 strain). At 10 µg/ml, poly(I) reduced binding of commercial killed Wood strain by 82% compared with control (p<.01) and had no effect on binding of other *S. aureus* forms (figure 4D). At 100 µg/ml poly(I), binding of commercial killed Wood strain was inhibited by 80% (p<.01), binding of RN6390 strain was partially inhibited (similar to our findings with the human macrophages, ∼45%, p<.05) and binding of live Wood and Cowan 1 strains was unaffected (figure 4D).

### Effect of signaling and cytoskeletal inhibitors on phagocytosis of unopsonized *S. aureus* strains

**Figure 7:**
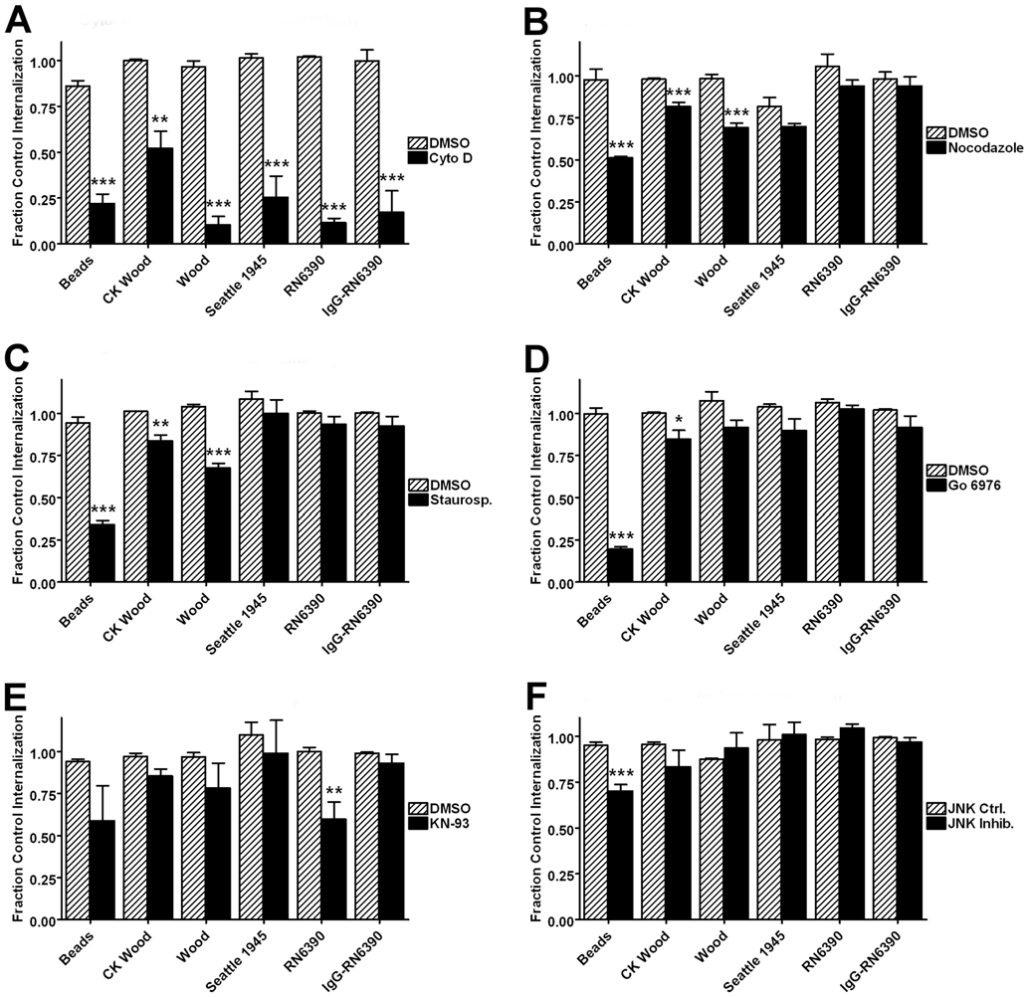
Effects of inhibitor panel on internalization of *S. aureus* strains. Adherent GM-MФ were treated with inhibitors or controls (table 1) prior to and during incubation with either fluorescent latex beads or *S. aureus*. Bars represent the controls as mean percent internalization of three normal donors±SEM. (A) Effect of actin polymerization inhibitor cytochalasin D. (B) Effect of microtubule assembly inhibitor nocodazole. (C) Effect of protein kinase inhibitor staurosporine. (D) Results of protein kinase C inhibitor Gö 6976. (E) Effect of c-Jun N-kinase inhibitor JNK inhibitor I. (F) Effect of CaM kinase II inhibitor KN-93. *p<0.05, **p<0.01, ***p<0.001 compared to both no treatment control and vehicle or inactive analog control.

**Figure 8:**
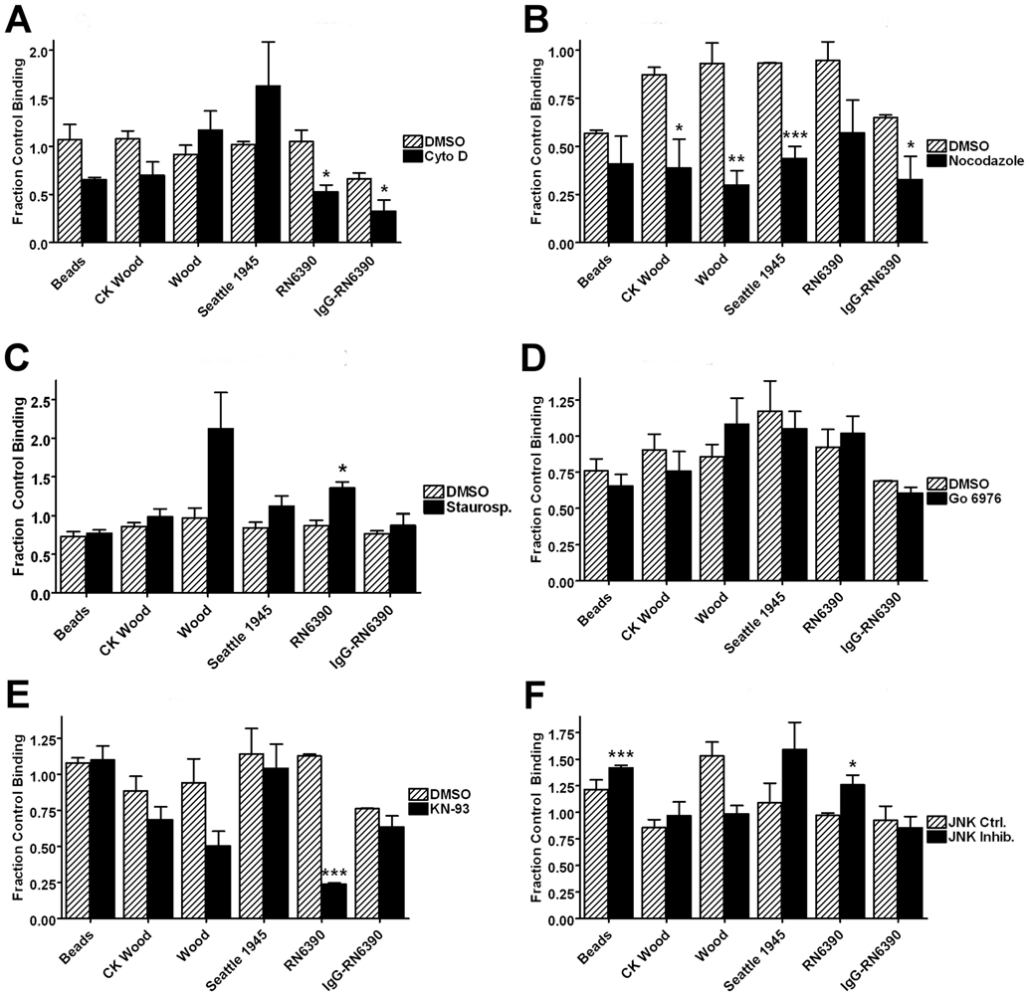
Effects of inhibitor panel on binding of *S. aureus* strains. Adherent GM-MФs were treated with inhibitors or controls (table 1) prior to and during incubation with either fluorescent latex beads or *S. aureus*. Bars represent the mean of the fraction of null control particles per cell of three normal donors±SEM. (A) Results of actin polymerization inhibitor cytochalasin D. (B) Results of microtubule assembly inhibitor nocodazole. (C) Results of protein kinase inhibitor staurosporine. (D) Results of protein kinase C inhibitor Gö 6976. (E) Results of c-Jun N-kinase inhibitor JNK inhibitor I. (F) Results of CaM kinase II inhibitor KN-93. *p<0.05, **p<0.01, ***p<0.001 compared to both null control and vehicle or inactive analog control.

To investigate the contribution of cytoskeleton and signaling pathways in phagocytosis of *S. aureus*, adherent GM-MФs were treated with a panel of inhibitors (table 1), or appropriate control vehicle solutions, prior to and during incubation with a panel of *S. aureus* strains as well as unopsonized latex beads, and analyzed by scanning cytometry as described in methods. The results are detailed in figures 7 and 8 and summarized in tables 3 and 4 show uniform effects of cytochalasin D, but extensive heterogeneity in the effects of other inhibitors among the strains tested.

**Table 3:** Summary of internalization inhibition results.

Inhibitor	Target	Beads	CK Wood	Wood	Seattle 1945	RN6390	IgG ops.RN6390
Poly(I) 10 µg/ml	SR binding	**-**	**-**	**-**	**-**	**-**	**-**
Poly(I) 100 µg/ml	SR binding	**nd**	**-**	**-**	**-**	**-**	**nd**
Cytochalasin D	actin	**+++**	**++**	**++++**	**++++**	**++++**	**++++**
Nocodazole	microtubules	**++**	**+**	**++**	**-**	**-**	**-**
Staurosporine	protein kinases	**+++**	**+**	**++**	**-**	**-**	**-**
KN-93	CaM Kinase II	**-**	**-**	**-**	**-**	**++**	**-**
Gö 6976	protein kinase C	**++++**	**+**	**-**	**-**	**-**	**-**
JNK Inhibitor I	c-Jun N-term kin.	**++**	**-**	**-**	**-**	**-**	**-**

Beads: biotinlyated carboxylated 1 micron green fluorescent latex beads. CK Wood: commercial killed AlexaFluor 488-conjugated *S. aureus* Wood strain. Wood: live S. aureus Wood strain. Seattle 1945: Live S. aureus Seattle 1945 strain. RN 6390: live GFP+S. aureus RN6390 strain. IgG ops. RN6390: IgG α-S. aureus opsonized RN6390.

+ <25% inhibition. ++25-50% inhibition. +++50-75% inhibition. ++++ >75% inhibition.

- not significantly changed. nd: not determined.

**Table 4:** Summary of binding inhibition results.

Inhibitor	Target	Beads	CK Wood	Wood	Seattle 1945	RN6390	IgG ops. RN6390
Poly(I) 10 µg/ml	SR binding	**+++**	**++++**	**-**	**-**	**-**	**-**
Poly(I) 100 µg/ml	SR binding	**nd**	**++++**	**-**	**-**	**++**	**nd**
Cytochalasin D	actin	**-**	**-**	**-**	**-**	**+++**	**++**
Nocodazole	microtubules	**-**	**++**	**+++**	**++**	**-**	**++**
Staurosporine	protein kinases	**-**	**-**	**-**	**-**	**^̂**	**-**
KN-93	CaM Kinase II	**-**	**-**	**-**	**-**	**++++**	**-**
Gö 6976	protein kinase C	**-**	**-**	**-**	**-**	**-**	**-**
JNK Inhibitor I	c-Jun N-term kin.	**^̂**	**-**	**-**	**-**	**^̂**	**-**

Beads: biotinlyated carboxylated 1 micron green fluorescent latex beads. CK Wood: commercial killed AlexaFluor 488-conjugated *S. aureus* Wood strain.

Wood: live S. aureus Wood strain. Seattle 1945: Live S. aureus Seattle 1945 strain. RN 6390: live GFP+S. aureus RN6390 strain. IgG ops. RN6390: IgG α-S. aureus opsonized RN6390.

+ <25% inhibition. ++25–50% inhibition. +++50–75% inhibition. ++++ >75% inhibition.

- not significantly changed. ^̂ 25–50% increased. nd: not determined.

Inhibition of actin polymerization by cytochalasin D (15 µM) strongly inhibited internalization of all *S. aureus* forms and latex beads (figure 7A). Nocodazole-mediated inhibition of microtubule formation reduced internalization of latex beads by 45% (p<.001), of commercial killed *S. aureus* Wood strain by 16% (p<.001) and of live Wood strain by 29% (p<.001), but had no significant effect on internalization of other unopsonized *S. aureus* strains or that of IgG-opsonized RN6390 strain (figure 7B).

Another example of heterogeneity can be seen in the pattern of inhibition resulting from staurosporine (1 µM) inhibition of protein kinase activity. Staurosporine blocked internalization of unopsonized beads by 59% (p<.001), commercial *S. aureus* killed Wood strain by 18% (p<.01) and live Wood strain by 29% (p<.001), but had no significant effect on internalization of other unopsonized strains of S. aureus or IgG-opsonized RN6390 (figure 7C).

Additional illustration of varied effects is seen in, inhibition of CaM kinase II by KN-93 (4 µM) treatment reduced both internalization (40%, p<.01) and binding (89%, p<.001) of unopsonized *S. aureus* RN6390 strain, but had no effect on phagocytosis of latex beads, other unopsonized *S. aureus* strains, or IgG-opsonized RN6390 (figure 7E and figure 8E).

## Discussion

We have developed and demonstrated the utility of a high throughput assays for quantifying phagocytosis of fluorescent or non-fluorescent bacteria by adherent macrophages in a 96-well microplate format. Assays of unopsonized latex bead and opsonized *S. aureus* RN6390 phagocytosis served as positive controls. Consistent with previous reports, binding of unopsonized beads was strongly blocked by the SR antagonist polyinosinic acid, and uptake of beads was significantly reduced by inhibition of actin (cytochalasin D) and tubulin (nocodazole) polymerization, protein kinases (staurosporine), protein kinase C (Gö 6976), and c-Jun N-terminal kinases (JNK). Also consistent with earlier studies, our assay showed that the uptake of IgG-opsonized (FcγR-mediated) uptake of *S. aureus* RN6390 was blocked by inhibition of cytochalasin D, but not by nocodazole or staurosporine.

Surprisingly, however, the classic SR inhibitor polyinosinic acid did not block binding of live *S. aureus* strains or of heat-killed *S. aureus* Wood strain, and a marked heterogeneity of inhibition patterns was found among strains and forms of *S. aureus* within the panel of inhibitors investigated.

A number of earlier studies have demonstrated the role of scavenger receptors, particularly SR-AI/II and MARCO, in phagocytosis of Gram-positive bacteria via the cell surface ligand lipoteichoic acid (LTA) [3], [5]–[12]. Many of these studies, including our own efforts, have utilized a commercial preparation of killed Wood strain bacteria [2], [4], [6], [8], [9]. Our results using this killed Wood strain in the experiments reported here are quite similar to prior findings. Binding by GM-MФs or adherent mouse peritoneal macrophages was almost completely abrogated by polyinosinic acid at 10 µg/ml. However, at the same concentration, polyinosinic acid did not block binding of live or heat-killed Wood strain, or live Seattle 1945, Cowan 1, or RN6390 strains, and only partially blocked binding of RN6390 strain at 100 µg/ml. These results suggest that SRs do not play a major role in the unopsonized phagocytosis of live or clinically relevant strains of *S. aureus*, and that the commercial killed Wood strain may be peculiar among *S. aureus* forms in its SR-mediated uptake. Although the panel of *S. aureus* strains studied here is far from comprehensive the consistency in this regard among those that were studied is noteworthy. The general concept of SR-mediated uptake of Gram-positive bacteria via their cell wall LTA, a known SR ligand, may require refinement. The data suggest that alternative receptors are likely involved. Indeed, a recent study of AM uptake of *Streptococcus pyogenes* revealed an ability of some pathogenic strains to evade SR-mediated uptake [38].

These findings are distinct from those of Thomas *et. al.* REF. They reported that SR-A deficient mice showed diminished clearance of live *S. aureus* Cowan 1 strain, as well as decreased survival, after i.p. injection. They also observed of impaired unopsonized phagocytosis of Cowan 1 strain *in vitro* by peritoneal macrophages from the same mice, and strong *in vitro* poly(I) inhibition of unopsonized Cowan 1 strain phagocytosis by peritoneal macrophages from wild-type mice [5]. One possible explanation for the discordance between our results and the *in vivo* results reported by Thomas *et. al.* is that mouse peritoneal macrophages might behave differently than human alveolar-like macrophages in this regard. Another possibility is that even though macrophage SR may not play a major role in binding of unopsonized S. aureus, SR binding may be facilitated after in vivo opsonization with serum or other components that are SR ligands. These speculations cannot, however, reconcile our *in vitro* poly(I) results with the Cowan 1 strain with those of Thomas et al., since we were unable to detect inhibition of binding by either human GM-MФs or wild-type mouse peritoneal macrophages. Hence, the basis for these differences remains unresolved.

A second surprising finding in our study was a remarkable variation among *S. aureus* strains in the effect of signaling inhibitors on uptake. Whereas inhibition of actin polymerization blocked uptake of all S. aureus strains and forms and inhibited binding of RN6390 strain only, blockade of microtubule assembly by nocodazole impaired internalization of the Wood strain (live or commercial killed) only and blocked binding of all but the RN6390 strain. Protein kinase inhibition by staurosporine inhibited uptake of live or commercial killed Wood strain, and PKC inhibition by Gö 6976 inhibited uptake of commercial killed Wood strain only. JNK inhibition had no effect on uptake of any strain or form of S. aureus. Finally CaM Kinase II inhibition by KN-93 blocked both binding and internalization of unopsonized RN6390 strain, but had no effect on phagocytosis of other strains. This heterogeneity of inhibition profiles among strains suggests that multiple signaling and cytoskeletal/mechanical process schemes for unopsonized phagocytosis exist and that the scheme used by the macrophage is determined by some as yet unknown variable strain-dependent characteristic of S. aureus.

We have used the high-throughput phagocytosis assay to investigate the effects of a relatively small panel of inhibitors on phagocytosis of a similarly small number of *S. aureus* strains. Further larger-scale studies utilizing more comprehensive inhibitor panels, or RNAi knockdown cell libraries are possible to more completely characterize the signaling pathways utilized by these and other *S. aureus* strains. The high-throughput scanning-cytometry based methods developed in the present study are well suited for these purposes. These techniques could also easily be adapted to study phagocytosis of other microorganisms by other cell types, and with the additional labeling of dead vs. live bacteria could be adapted to allow study of both phagocytosis and killing of pathogens.
